# Rapid correction of severe hyponatremia after hysteroscopic surgery – a case report

**DOI:** 10.1186/s12871-015-0070-4

**Published:** 2015-06-09

**Authors:** Philip Hepp, Tobias Jüttner, Ines Beyer, Tanja Fehm, Wolfgang Janni, Enrico Monaca

**Affiliations:** 1 Clinic for gynecology and obstetrics, Heinrich-Heine-University, Moorenstr. 5, 40225 Düsseldorf, North Rhine-Westphalia Germany; 2 Clinic for anesthesiology, Klinikum Wolfenbüttel, Wolfenbüttel, Lower Saxony Germany; 3 Clinic for gynecology and obstetrics, University Ulm, Ulm, Baden-Württemberg Germany; 4 Clinic for anesthesiology, Heinrich-Heine-University, Düsseldorf, North Rhine-Westphalia Germany

**Keywords:** Acute hyponatremia, TURP, Hysteroscopy, Myoma, Gynecology

## Abstract

**Background:**

One of the most feared complications during hysteroscopic surgery is haemodilution by absorption of distension media. One facet of haemodilution, i.e. hyponatremia, can lead to respiratory distress, pulmonary oedema, as well as cardiovascular collapse.

**Case presentation:**

Here we report the swift recovery of a 45 year, female, Caucasian patient with acute hyponatremia (74 mEq/L) and pulmonary oedema by the employment of rapid correctional strategies.

**Conclusion:**

The absorption of irrigation fluids, as presented in this case, is an inevitable side effect of hysteroscopic surgery. Utmost caution should, therefore, be mandatory to reduce and actively monitor fluid intake. If these measures fail, as in the case presented here, it is essential to rapidly eliminate any free water and to normalize the sodium levels. Anecdotal reports of pontine myelinolysis are not in line with literature concerning acute hyponatremia and should, therefore, not obstruct determined action against it.

## Background

Operative hysteroscopy is a safe surgical approach to the uterus with complications reported in only 0.24 % - 2 % of cases [1]. One of the most feared complications during this procedure is severe haemodilution by the absorption of the distension medium. This phenomenon was first described during the endoscopy of patients undergoing transurethral prostatectomy [2]. Due to this, it is often referred to in the literature as Trans-urethral resection of the prostate syndrome (TURP-Syndrome). Despite this fact, the prevalence of haemodilution during hysteroscopic surgery seems to be even higher than during transurethral prostatectomies [3]. The first symptoms of an electrolytic imbalance are nausea and vomiting, visual disturbances, and altered states of consciousness [4]. However, hysteroscopic surgery is often performed with general anaesthesia and early symptoms can therefore be masked. Excessive fluid uptake presents itself during surgery by advanced symptoms, such as respiratory distress, pulmonary oedema and cardiovascular collapse due to fluid overload, or seizures and encephalopathy due to acute hyponatremia [5]. In addition, there are cases of symptomatic hyponatremia starting up to 24 h after surgery, i.e. at a time where patients are typically already released from hospital [6]. Only few studies are available concerning the prevention of intravasation during hysteroscopy, as well as the treatment of fluid overload and hyponatremia [7]. Here we report on a patient undergoing a hysteroscopic resection of a submucosal leiomyoma and concomitant endometrial ablation, who, due to excessive distension medium uptake, developed critical acute iatrogenic hyponatremia (74 mEq/L) with severe pulmonary oedema.

## Case presentation

A 45-year-old, 58 kg, 156 cm woman presented with increased and prolonged vaginal bleedings. A physical examination and an ultrasound showed a submucosal leiomyoma of the uterus. There were no other symptoms or pathological findings. Preoperative blood tests, which included electrolytes analyses, a full blood exam, and coagulation parameters, showed no anomalies. Most notably, both creatinine and sodium were within normal levels, i.e. 0.7 mg/dL and 137 mEq/L, respectively. The patient had a history of encephalopathy during childhood of an unknown origin without sequelae, an elective caesarean section (1997) due to leiomyoma, laparoscopic myomectomy and removal of an endometrial cyst of the ovary (1999), and a second caesarean section (2000) due to an uterine instability subsequent to the first caesarean section and myomectomy. The patient had no known allergies. She was taking daily doses of ethinyl estradiol (0.02 mg) and levonorgestrel (0.1 mg) continuously over a period of six months. The patient refused a hysterectomy but an endoscopic removal of the leiomyoma and an endometrial ablation was decided on, subsequent to which the patient gave written informed consent. The patient was administered with 7.5 mg midazolam orally 60 min preoperatively. Subsequent to placing the patient in the lithotomy position, the application of a peripheral venous catheter and the attachment of standard monitoring equipment (electrocardiogram, non-invasive blood pressure cuff and a peripheral capillary oxygen saturation analyzer), anaesthesia was induced intravenously with propofol (2 mg/kg) and sufentanil (0.3 μg/kg).

**Fig. 1:**
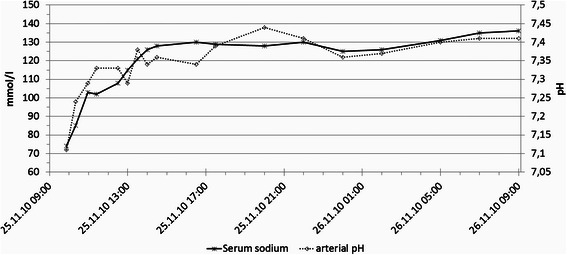
Graphic illustration of sodium and pH level until recovery

A laryngeal mask was inserted, and after the correct positioning was verified, the lungs were ventilated with a fraction of inspired oxygen (FiO2) of 0.5 and a minute volume (MV) of 74 mL/min/kg at a ventilation frequency of 10/min in volume controlled mode (IPPV). The positive end-expiratory pressure (PEEP) level was 0 Pa and the peak pressure was 900 Pa. Anaesthesia was maintained with propofol (8 mg/kg/h). After anaesthesiological clearance the endoscopy commenced. Purisole® SM (Fresenius Kabi Inc., Germany) was utilized as the distension medium. This standard hypotonic solution contains 27 g sorbitol and 5.4 g mannitol per liter. A pressure of 16 kPa (120 mmHg) was applied to unfold the uterine cavity. Endometrial ablation and dissection of the submucosal myoma were performed within 70 min by monopolar resection. The duration of the procedure was prolonged due to the reduced visibility caused by excessive bleeding and cervical leakage of distension medium, which impeded proper expansion of the uterine cavity. The ventilation pressure increased slowly and continuously 60 min subsequent to the start of surgery. The compliance of the lungs, as well as the achievable minute volume within the acceptable airway pressure limit of 2 kPa, decreased. It was presumed that the level of anaesthesia was insufficient and was followed by the increased administration of propofol (10 mg/kg/h). The additional inhalation of fenoterol did, however, not improve the situation. As ventilation continued to be difficult, the laryngeal mask was replaced by a 7.0 cm (inside diameter) cuffed tube subsequent to the administration of 100 mg succinylcholine as a muscle relaxant. Approximately 50 mL of a foamy, transparent liquid was aspirated from the trachea. Testing with litmus paper indicated an alkaline pH and, therefore, not the aspiration of gastric content. The amount of the pulmonary oedema increased and, despite high levels of peak airway pressures at this point approximately 3 kPa (= set peak pressure limit), adequate ventilation was not possible. Blood gas and electrolyte analyses indicated acidosis, as well as a severe hyponatremia (pH 7.11; Na 74 mEq/L; partial pressure of CO_2_ 49.9 mmHg; saturation of O_2_ 97 % at FiO_2_ 1.0). Cardiopulmonary changes remained minimal. 40 mg of furosemide and 500 mL of 3 % NaCl solution were immediately administered at an infusion rate of 500 mL/h, with constant monitoring for electrolyte changes. As the patient showed clinical signs of impaired haemostasis, the operation procedure was stopped. The patient was admitted to the intensive care unit with continuous administration of 3 % NaCl at a rate of 50 mL/h and electrolyte control. Plasma sodium levels increased from 74 mEq/L to 103 mEq/L after one hour, and to 130 mEq/L after 7 h (Fig. 1). All together 800 mL of 3 % NaCl solution were administered within 7 h totalling at 24 g. Subsequently, sodium replenishment was stopped and the patient was extubated without problems. Immediate neurological assessment revealed no detectable deficits. 20 h after the onset of hyponatremia, the patient’s sodium levels returned to within the normal range. Until then, the cumulative renal excretion was 8.5 L. 9 L of distension medium was used intraoperatively with a calculated uptake of 5 L. The patient was relocated to the gynaecologic ward the following day and was released from the hospital on the third postoperative day without any detectable sequelae.

## Discussion

The absorption of the distension medium, as presented in this case, is an inevitable side effect of vessel damage and the increased intrauterine fluid pressure due to the opening of the uterine wall during hysteroscopy [8]. Utmost caution should, therefore, be observed to reduce and monitor fluid intake. Various aspects should be taken into account in order to maintain patient safety throughout the procedure. The 2013 edition of the American Association of Gynecological Laparascopists Practice Guidelines advise the choice of a distension medium that is the least likely to cause complications in the event of excessive absorption; minimizing the systemic absorption of the distension medium during surgery; and early recognition of excessive absorption [7].

The choice of the distension medium is limited by the type of the hysteroscope used. In hysteroscopes working with monopolar electrosurgery, only electrolyte free liquids may be employed, whereas bipolar instruments allow for the use of isoosmotic, electrolyte balanced media, such as saline 9 %. In this case we used a monopolar hysteroscope in combination with the very common mixture of 0.5 % mannitol and 3 % sorbitol (Purisole® SM) as distension medium, which is electrolyte free and hypoosmotic (178 mOsm/L). This accounts for the decreased sodium level and fluid overload in our patient. Despite the fact that isotonic saline can also lead to a fluid overload and consequent right-sided heart failure [9, 10], it precludes an electrolyte imbalance and therefore seems to be the safer choice [11].

In this surgery 16 kPa (120 mmHg) distension medium pressure (DMP) was applied. When the DMP is above the mean arterial pressure (MAP), the risk for fluid overload and hyponatremia is significantly increased [12]. This is supported by data from a retrospective study, which indicated that the amount of fluid absorbed, does not increase proportionally to the DMP. Instead, there seems to be virtually no intravasation if the DMP is below the MAP, whereas significant intravasation is common in patients where the DMP exceeds the MAP [13].

As stated before, the type of surgery, as well as the amount of myometrial damage inflicted, plays a crucial role in the incurrence of fluid overload. Reported data from hysteroscopic resectioning of leiomyomas point towards a close relation between the water intake and the size and intramural portion of the resected leiomyoma [14]. Risk assessment can, thus, be made by a concise preoperative estimation of the expected endometrial or myometrial damage.

In addition to the above, a crucial requirement for volume shift is time. Even though reports exist of clinically overt hyponatremia and fluid overload occurring as early as 15 min after the commencement of surgery [15], most publications report that the first signs of hyponatremia only occur after more than 60 min (Table 1). Operation time should therefore be confined to 60 min or, if that is not possible, even more attention should be paid to the calculated water uptake.

**Table 1:** Overview of reported cases with severe hyponatremia during hysteroscopic surgery

Case report	Lowest Na^+^	Normal Na^+^ after	est. Fluid absorption	Irrigation fluid used	Max. fluid pressure	Surgery time	Treatment for hyponatremia	First clinical manifestation
S. Almonti et al. 2013 [34]	120 mmol/L	10 h	2 L	E-free Gycin 1.5 %	na	60 min	7 % NaCl; Furosemid; Mannitol	Myoclonus
M. Yaprak et al. 2013 [27]	99 mmol/L	14 h	5 L	Mannitol 5 %	na	na	3 % NaCl; Furosemid	Altered mental status
N. Sethi et al. 2012 [33]	100 mmol/L	na	1 L	Glycin 1.5 %	80 mmHg	45 min	3 % NaCl; Furosemid; sodium bicarbonate	Hypoxia
B. Yang et al. 2012 [19]	120 mmol/L	12 h	3 L	Dextrose 5 %	150 mmHg	70 min	3 % NaCl; Furosemid	Flatulence and coma
Y. Woo et al. 2011 [21]	87 mmol/L	48 h	24 L	2.7 % sorbitol and 0.54 % mannitol	150 mmHg	60 min	3 % NaCl; Furosemid	Increased ventilation pressure
G. Lee et al. 2009 [35]	89 mmol/L	24 h	7 L	2.7 % sorbitol and 0.54 % mannitol	100 mmHg	40 min	2 % NaCl; sodium bicarbonate; Furosemid;	Hypotonia, bleeding leading to hysterectomy
G. Serocki et al. 2009 [36]	106 mmol/L	24 h	0.4 L - 4 L	2.7 % sorbitol and 0.54 % mannitol	180 mmHg	25 min	0.9 % NaCl	Hypoxia
C. Estes et al. 2003 [37]	122 mmol/L	8 h	2.4 L	Glycin 1.5 %	na	90 min	Furosemid	na

A precise monitoring of fluid in- and outtake is also mandatory to allow the surgeon to decide when to terminate the procedure before the intravasation elicits a symptomatic response. There are different approaches to estimate the patient’s intake of distension medium, the simplest being the subtraction of the fluid outflow from the inflow. Different factors, however, make this strategy prone to failure. First of all, the collection of all fluids returning from the patient can be challenging. Due to insufficient cervical closure, as in this case, a significant amount of fluid can be spilled, despite the use of collection bags. Furthermore, the actual volume of distension fluid per bag exceeds the declared volume by approximately 2.8 % to 10 % [16, 17]. Additionally, the determination of the actual volume left in a used bag is a source of error [16]. To account for this issue, weight-based infusion systems are available. Through the continuous measurement and comparison of the weight of infusion bags, as well as the collected returning liquids, a much more reliable assessment of the amount of intravasation can be acquired. Nevertheless, every surveillance system will fail if the user neglects the warnings given. In our case, the indicated loss of water was attributed to the intensive spilling of water onto the drapes and the floor, which has been frequently reported as a confounding cause for volume overload during hysteroscopy [18–21].

As a consequence of this case we altered our management of patients scheduled for hysteroscopic surgery. First of all, we use bipolar surgery with isotonic distension fluid whenever possible. Additionally, we appointed a second nurse in the operation theatre for keeping track of in- and outflow, as well as providing regular feedback to the surgeon and anaesthesiologist for every 100 mL uptake.

If monitoring fails leading to the development of volume overload and hyponatremia, the rapid commencement of the appropriate treatment is imperative. Existing data indicates that patients who suffer from acute symptomatic hyponatremia for whatever reason, have a mortality rate of up to 40 % [22, 23]. Acute hyponatremia, in this context, is defined as hyponatremia lasting less than 48 h [24]. The pathophysiology and the clinical presentation of acute and chronic hyponatremia differ greatly. Chronic hyponatremia is often clinically less perceptible and its too rapid correction can lead to severe neurological damage, including central pontine myelinolysis, pseudobulbar palsy and quadriparesis [25, 26]. To date, no such event has been described for the rapid treatment of acute hyponatremia [27]. If anything, the shortening of the hyponatremic time span seems to decrease the risk of cerebral oedema and pontine myelysis [21, 28].

Unfortunately, the recently published guidelines for the rapid correction of severe symptomatic acute hyponatremia were not yet released at the time of the event reported here. Contrary to these guidelines, NaCl was administered as a continuous infusion, whereas the guidelines favour the bolus administration of 150 mL 3 % NaCl or 2 mL/kg. This should be repeated every 20 min until a significant change in the sodium level occurs. The patient received a total of 500 mL of 3 % NaCl in 1 h, resulting in the amount as recommended by the guidelines [29, 30]. The infusion rate was subsequently reduced to 50 mL/h for six more hours to avoid overcorrection.

To date, no data from controlled trials exists, which examine the best, or the maximal, correction rate in acute iatrogenic isotonic hyponatremia. A correction rate of 25 mEq/L is generally recommended within 48 h until the sodium in the serum is above 120 mEq/L, but without reaching normal or hypernatremic levels [31, 32]. Despite this, most publications report significantly higher correction rates [18, 20, 21, 33], even correcting as rapidly as the initial sodium level decreased [27]. Encephalopathy or pontine myelysis did not occur in any of these cases and patients were reported to have recovered fully after therapy. An overview of reported cases in the literature with severe hyponatremia during hysteroscopic surgery is provided in Table 1. None of these publications reported a detrimental effect associated with the rapid correction of hyponatremia. More so, all patients recovered fully, even in cases with very low sodium levels.

## Conclusion

The absorption of irrigation fluids, as presented in this case, is an inevitable side effect of hysteroscopic surgery. Utmost caution should, therefore, be mandatory to reduce and actively monitor fluid intake. If these measures fail, it is essential to rapidly eliminate any free water and to normalize the sodium levels. Anecdotal reports of pontine myelinolysis are not in line with literature concerning acute hyponatremia and should, therefore, not obstruct determined action against it.

In conclusion, our case strengthens the value of the rapid correction of acute hyponatremia.

## Consent

Written informed consent was obtained from the patient for publication of this Case report and any accompanying images. A copy of the written consent is available for review by the Editor of this journal.
